# Increasing condom use in heterosexual men: development of a theory-based interactive digital intervention

**DOI:** 10.1007/s13142-015-0338-8

**Published:** 2015-08-13

**Authors:** R. Webster, S. Michie, C. Estcourt, M. Gerressu, J. V. Bailey, on behalf of the MenSS Trial Group

**Affiliations:** 1 eHealth Unit, Research Department of Primary Care and Population Health, University College London, Royal Free Hospital, Rowland Hill Street, London, NW3 2PF UK; 2 Research Department of Clinical, Educational, and Health Psychology, University College London, London, UK; 3 BICMS, Barts and The London School of Medicine & Dentistry, Barts Sexual Health Centre, Queen Mary University of London, St Bartholomew’s Hospital, London, UK; 4 Department of Infection and Population Health, University College London, London, UK

**Keywords:** Behaviour change, Sexual health, Intervention development, Theory, Internet

## Abstract

Increasing condom use to prevent sexually transmitted infections is a key public health goal. Interventions are more likely to be effective if they are theory- and evidence-based. The Behaviour Change Wheel (BCW) provides a framework for intervention development. To provide an example of how the BCW was used to develop an intervention to increase condom use in heterosexual men (the MenSS website), the steps of the BCW intervention development process were followed, incorporating evidence from the research literature and views of experts and the target population. Capability (e.g. knowledge) and motivation (e.g. beliefs about pleasure) were identified as important targets of the intervention. We devised ways to address each intervention target, including selecting interactive features and behaviour change techniques. The BCW provides a useful framework for integrating sources of evidence to inform intervention content and deciding which influences on behaviour to target.

## BACKGROUND

### The problem

Sexually transmitted infections (STIs) are a major public health problem, with high social and economic costs [1]. There were 450,000 STI diagnoses reported in England in 2012, an increase of 5 % over the previous year [2]. Condoms are effective for prevention of STIs; however, there are many barriers to successful use; for example, decrease in sensation, interruption of sex, incorrect size or fit, and the use of alcohol/recreational drugs [3, 4]. Men are more likely to report experiencing decreased sensation with condoms [3], which is often used as a reason to try and dissuade a partner from using condoms [5], and they may also have more power to influence condom use for penetrative sex [6, 7], making them an important target group for prevention efforts [3]. However, men are less likely than women to visit health professionals and generally have shorter clinic appointments [8, 9] and are also less likely to receive consultations regarding contraceptive choices. As a result, they may be less likely to be offered health promotional advice or risk reduction counselling in the context of routine appointments. An interactive digital intervention (IDI) therefore offers an alternative and additional avenue to reach men who are not accessing face-to-face health services.

### Interactive digital interventions for sexual health

Interactive digital interventions (IDIs) are defined as ‘Computer-based programmes that provide information and one or more of: decision support, behaviour change support, or emotional support for health issues’ [10]. IDIs can offer personally relevant tailored material and feedback [11], and delivery via the web offers anonymous access [12], along with minimal staff time and training requirements to deliver. IDIs have been shown to have a moderate impact on condom use (*d* = 0.259; 95% CI 0.201–0.317) [13], as well as increasing knowledge regarding sexual health, self-efficacy for safer sex behaviours, and safer sex intention [10, 13, 14].

Interventions are more likely to be effective if they are grounded in theory [15] and involve a high level of user input in the development process [16]. The MenSS (Men’s Safer Sex) website was therefore a theory- and evidence-based IDI, developed with a high level of user input, aimed at increasing condom use in men who have sex with women. This paper outlines the development process of the MenSS website, thus providing transparency for readers, and an example of how an evidence- and theory-based IDI may be developed.

### Intervention development

**Fig. 1:**
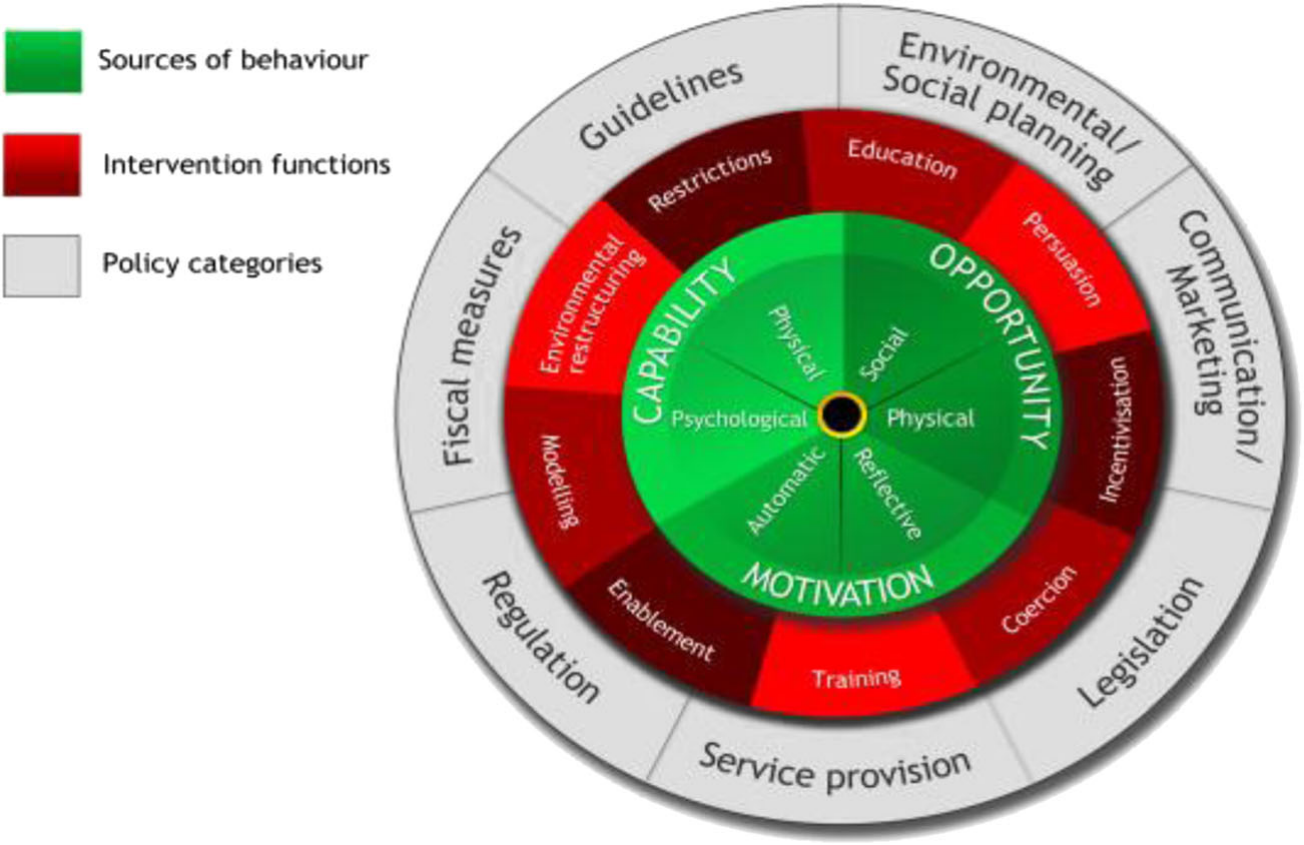
The Behaviour Change Wheel; Michie et al. [20]

Understanding the exact nature and content of complex behaviour change interventions is important for understanding which intervention components are the ‘active ingredients’ [17]; however, intervention designs are frequently not well described in publications [18]. As a result, replication is difficult, and future intervention designers cannot learn from what is and is not effective [18, 19]. Lack of detail in the description of the content and development process for an intervention limits the evaluation of its quality or the possibility of linking content to outcome. A further issue is that even if one is aware that theory and user involvement should be incorporated into intervention development, it can be difficult to know exactly how to achieve this. The Behaviour Change Wheel (BCW, Fig. 1) [20] is one method that can be used to guide this process. It provides a framework for the process of developing complex interventions. It represents a synthesis of 19 theoretical frameworks of behaviour change pathways. It has the advantage of being comprehensive (i.e. it covers all the main intervention functions and policy categories), coherent (i.e. categories within it are consistent, and it provides systematic steps for intervention design), and being linked to an overarching model of behaviour. The BCW has been used to develop interventions that were both acceptable to users and effective in achieving their aims [21–23]. The main steps are as follows^1^:

Step 1: Specifying the target behaviour and population
  This involves specifying who needs to do what, where, when, and how often. Having a specific target behaviour allows for a focussed development process and facilitates decisions regarding appropriate measurable outcomes [24]. A well-defined target population enables the intervention developer to select an appropriate platform and tailor the content to the users’ specific needs and preferences.
Step 2: Identifying theoretical domains that explain the behaviour
  At the heart of the BCW is a model in which behaviour change is conceptualised as requiring a shift in capability, opportunity and/or motivation, with these three drivers being part of an interacting system (the COM-B model (capability, opportunity, motivation, behaviour), see Fig. 1). Capability may be physical or psychological; opportunity may be physical or social; motivation may be reflective or automatic. These subdivisions can be further elaborated into 14 detailed theoretical domains, specified by the Theoretical Domains Framework (TDF, see Table 1) [25]. The TDF is an amalgamation of 128 theoretical constructs from 33 theories of behaviour and behaviour change. It can be used to conduct a theoretically based assessment of the problem [26, 27], which identifies mechanisms of action to be targeted by the intervention. The advantage of using the TDF over a single theory of health behaviour is that it delineates multiple distinct explanatory domains, which may be more appropriate for the development of a complex intervention, since a greater number of potential influences on behaviour can be considered.

**Table 1:** Explanatory domains of the Theoretical Domains Framework, categorised by COM-B dimensions [25]

COM-B component		TDF domain
Capability	Psychological	Knowledge
		Cognitive and interpersonal skills
		Memory, attention, and decision processes
		Behavioural regulation
	Physical	Skills
Opportunity	Social	Social influences
	Physical	Environmental context and resources
Motivation	Reflective	Social/professional role and identity
		Beliefs about capabilities
		Optimism
		Beliefs about consequences
		Intentions
		Goals
	Automatic	Reinforcement
		Emotion
Step 3: Identifying how explanatory domains should be targeted
  The BCW outlines nine possible ‘intervention functions’ which may be utilised: education, persuasion, incentivisation, coercion, training, restriction, environmental restructuring, modelling, and enablement. These delineate what ‘function’ the intervention will serve (i.e. to educate people, to train people, to coerce people), and appropriate functions are recommended for each of the explanatory domains identified in step 2 [20, 24]. At this point, it is important to consider which methods will be most appropriate for the intervention given its context i.e. target population and setting (e.g. in terms of acceptability, affordability, practicability) [24].
Step 4: Selecting standardised behaviour change techniques
  Once intervention functions have been identified, it is possible to identify the standardised behaviour change techniques (BCTs) which are relevant to each function. A BCT is an active component of an intervention designed to change behaviour [28] and is applicable to a range of health behaviours [29]. One can therefore select BCTs, considering the appropriateness for the population, setting, and intervention format. This approach has been used successfully in a range of interventions varying in mode of delivery, content, target behaviour, and context [19, 21, 30].

## AIM

The purpose of this study is to describe the development process of an IDI to increase condom use (the Men’s Safer Sex (MenSS) website, https://www.menss.co.uk) and to demonstrate the utility of the BCW as a framework for this process through a practical example.

## METHODS

Three sources of data were used to inform the intervention development process: the research literature, expert views, and interviews with the target population (men in sexual health clinics). The BCW and the TDF were used as frameworks to guide the analysis and synthesis of the evidence. Ethical approval was provided by the London–City and East NHS Research Ethics Committee (reference number 13/LO/1801). The intervention website was developed by a software development company (Digital Life Sciences; http://www.digitallifesciences.co.uk/).

### Data collection

#### Research literature

A targeted literature review identified research on men’s barriers and facilitators to condom use. Search terms included ‘men’, ‘heterosexual’, ‘condom’, and ‘barriers’, and articles were selected which reported risk factors for non-condom use, theoretical correlates of condom use, and men’s views and experience of using condoms. The full text of included studies [3, 31–66] were summarised and synthesised.

#### Expert consultation

Two expert workshops were held. Attendees at the first day-long workshop included 13 experts in the area of men’s sexual health and/or behaviour change, including sexual health clinicians, health advisors, and academic professors. The workshop was structured around the BCW, asking participants to give their opinions about the explanatory domains, intervention functions, and the format of the intervention.

A second (half-day) workshop was held following interviews with male clinic attenders, to guide final decisions regarding the intervention design and content. This workshop included five experts in the fields of sexual health, sex education, and web development. Participants were presented with the findings from the development process and asked to prioritise aspects of intervention content. This informed decisions regarding resource allocation during the creative process of designing intervention features. For example, while interactive features were considered the ideal, budget constraints meant that we could not develop interactive features to target every influence on behaviour. Therefore, some ‘lower priority’ influences on behaviour (or those for which interactive features were deemed inappropriate, e.g. sexual pleasure) were targeted with written information.

#### Interviews with the target population

Semi-structured qualitative interviews were conducted with the potential target population: 20 men who visited two sexual health clinics in central London (mean age = 31, range 20–52; 17 men currently sexually active with women, three with men). Interviews focussed on barriers to and facilitators of condom use, potential intervention design, and content and mode of delivery. The recordings were listened to, themes summarised, and then analysed using qualitative thematic content analysis [67].

#### User testing

Focus groups (*N* = 3) were held with male sexual health clinic users (*N* = 16) to refine the MenSS website content and design. After each focus group, themes were summarised and content and design changes were made in response to the feedback. Once an initial version of the website was ready, interviews were conducted with male sexual health clinic users (*N* = 7) to ensure acceptability, relevance, and understanding, and to identify any bugs or usability issues. Findings fed into the final version of the intervention.

## INTERVENTION DEVELOPMENT: PROCESSES AND OUTCOMES

The following section outlines the methods (processes) and results (outcomes) for each step of the intervention development process. Full details regarding the content of the intervention can be found in Webster et al. [68].

### Step 1: specifying the target behaviour and population

#### Process

The target behaviour and population were discussed at the initial expert workshop. Final decisions regarding this were made by the study team.

#### Outcome

Condom use was pre-defined as the specified target behaviour. Men who have sex with men (MSM) were identified in the expert workshop as a high-risk group for STI; however, the research team identified a number of existing interventions for MSM and a lack of interventions for men who have sex with women [69, 70], so it was therefore decided that the intervention would target men who have sex with women. Men in sexual health clinic settings may be at higher risk of repeat sexually transmitted infection [71], and so it was decided that the intervention would be targeted at men in clinic settings.

The target behaviour and population also influenced the choice of intervention format (i.e. an interactive website or a smartphone application). Technology usage data suggested high smartphone usage in the target population; however, it was difficult to select a platform (e.g. Android, iOS) that was used by the majority of the population and the budget precluded developing the application for more than one platform. Issues of privacy or lack of motivation were also a concern, potentially preventing users from downloading an application about condoms to their smartphone. As a result, it was decided that the intervention would take the form of an interactive website, rather than a smartphone application.

### Step 2: Identifying theoretical domains that explain the behaviour

#### Process

In order to determine which domains should be targeted within the intervention, data from the interviews, workshops, and literature review were organised into the categories of the COM-B and TDF (see Table 1). Data were coded using the TDF as a coding frame; initially for each data source, then findings were synthesised. Domains were prioritised in order of importance to guide decisions regarding what to target with the website, considering available resources. Domains were considered important if they were strongly supported by all three data sources. Where evidence from the data sources was conflicting, the research team discussed each case and made a decision regarding whether it should be targeted, and how much of a priority it should be, drawing on their knowledge and experience of the field.

#### Outcome

Of the 14 domains in the TDF, 12 were deemed to be important and were therefore targeted by the intervention: skills, knowledge, cognitive and interpersonal skills, social/professional role and identity, beliefs about capabilities, beliefs about consequences, goals, intention, memory, attention, decision processes, emotion, environmental context and resources, and social influence (see Table 2).

**Table 2:** BCTs used in each intervention component

Domain	Findings	Intervention functions	Behaviour Change Techniques
Skills	High rate of errors in condom use [58]	Training	Instruction on how to perform the behaviour; demonstration of the behaviour
Knowledge	Men lacked knowledge about condom sizes and types and how they may improve pleasure and comfort (interviews)	Enablement	Problem solving
	Men had incorrect knowledge about risk of contracting STIs (interviews); knowledge about risk is related to condom use [48]	Education	Information about health consequences; vicarious consequences
Cognitive and interpersonal skills	Communication related to use of condoms [48]; difficulty knowing when to suggest condom use (interviews); fear that partner may be offended [51]; difficulty negotiating if partner is reluctant (interviews)	Education, training, persuasion, enablement	Instruction on how to perform the behaviour; information about social and environmental consequences; information about others’ approval; information about health consequences; verbal persuasion about capability
Memory, attention, and decision processes	Being caught in the ‘heat of the moment’ leads to non-condom use (due to high level of arousal and lust, and competing desire for increased pleasure) (interviews) [35]	Enablement, education, training, environmental restructuring	Problem solving; verbal persuasion about capability; information about health consequences; instruction on how to perform the behaviour; information about antecedents; restructuring the physical environment; anticipated regret; mental rehearsal of successful performance
Emotion			
Social/professional role and identity	Self-concept and values are related to condom use [43, 51], e.g. fostering/reinforcing being ‘responsible person’, who cares about others’ health, may be a facilitator to condom use	Persuasion	Information about others’ approval
Beliefs about capabilities	Literature suggested this may be an important predictor of behaviour [48, 59]	Enablement	Verbal persuasion about capability; mental rehearsal of successful performance
Beliefs about consequences	Perceptions that condoms negatively impact on pleasure and intimacy related to non-use (interviews) [3, 60–65]	Persuasion, enablement, education, incentivisation, training, environmental restructuring	Non-specific incentive; restructuring the physical environment; instructions on how to perform the behaviour; behaviour substitution; information about health consequences; focus on past success, distraction; behavioural practice/rehearsal; anticipated regret; information about social and environmental consequences; social incentive
	Belief that STIs do not have negative consequences for men (interviews)	Education	Information about health consequences
Intentions	Literature suggested this may be an important predictor of behaviour. [48, 66]	The aim of the intervention as a whole was to increase intention. Specific intervention functions and BCTs not identified.	
Goals	Goal setting may be an important tool to implement advice in the intervention. [77, 78]	Enablement	Goal setting; action planning; review behaviour goals
Environmental context and resources	Intoxication due to alcohol or recreational drugs often prevents condom use (interviews) [31]	Enablement, education, incentivisation	Problem solving; verbal persuasion about capability; information about social and environmental consequences; information about antecedents; anticipated regret; non-specific incentive
Social influence	Men generally had a desire for a good reputation for sexual performance, and their partner’s opinions held a lot of importance (interviews) [51]	Persuasion, education, incentivisation	Information about others’ approval; social incentive

The most prominent domains were beliefs about consequences (impact on pleasure), knowledge (about risk of STIs and about condom sizes and types), memory, attention, and decision processes/emotion (difficulty using condoms in the ‘heat of the moment’). Psychological capability and reflective and automatic motivation therefore were found to be important targets for change for this population.

Some domains contained one or more sub-themes. ‘Beliefs about consequences’ incorporated both beliefs about the impact of condoms on sexual pleasure and the belief that STIs were not serious and did not have negative long-term consequences for men. A prominent aspect of knowledge was knowledge about risk of STI—men often felt that they could judge the risk of a situation (e.g. by a partner’s appearance or whether the partner was in a relationship). In addition, a lack of knowledge regarding condom sizes and types and how they might impact positively on sexual pleasure was identified. Some barriers to condom use fell into more than one domain; for example, being caught in the ‘heat of the moment’ was considered to come under ‘emotion’, as it relates to lust. However, this emotion also interfered with memory, attention, and decision processes.

For some domains, the sources of evidence were inconsistent. For example, both experts and interview participants considered that men possessed the skills in applying condoms; however, evidence from the literature suggests that rates of errors in condom use are high (e.g. putting it on the wrong way then turning it over, failing to squeeze air from the tip). The research team therefore decided that it was important to target this domain.

### Step 3: identifying how explanatory domains should be targeted

#### Process

Once appropriate domains had been selected, intervention functions were then identified by reviewing the possibilities for each domain as defined in the BCW guide [24] and considering which functions were likely to be most effective given the fieldwork findings. Consideration was given to the preferences outlined by participants in the interviews and what would be feasible and affordable (in terms of software development) to include.

#### Outcome

A full outline of the intervention functions selected for each domain can be found in Table 2. For the majority of domains, the intervention functions were very straightforward to select. For example, ‘skills’ lends itself to ‘training’, and ‘knowledge’ lends itself to ‘education’. Persuasion and enablement were used in a number of sections of the website, to target motivation. Coercion and restriction were deemed to be inappropriate, unfeasible, and potentially unacceptable to participants.

### Step 4: selecting standardised behaviour change techniques

#### Process

A number of potential BCTs appropriate for each intervention domain and function were identified using the BCW guide [24] [29]. These were reviewed for appropriateness to the population and intervention format, and a list of proposed BCTs for each domain was created. The selected BCTs were translated into the website intervention features and activities in a creative process involving the research team and the software development company, who also ensured the that ideas were feasible and affordable. Website content was then reviewed in comparison to the BCT taxonomy [29] to confirm that activities and wording met the standardised criteria for each proposed BCT.

#### Outcome

Full information regarding intervention content and how BCTs were operationalised can be found in Webster et al. [68]. For some domains, one particular BCT was focussed on. For example, problem solving was identified as an important way to target knowledge about condoms (and how they may impact on problems such as lack of pleasure), and a tailored feedback activity was developed based around this one BCT. For knowledge regarding STI risk, the BCTs ‘information about health consequences’ and ‘vicarious consequences’ were selected and operationalised in a quiz-based activity and a click-through activity demonstrating the consequences of unprotected sex. ‘Instruction on how to perform the behaviour’ and ‘demonstration of the behaviour’ were selected as the simplest ways of giving training and education about condom use skills and were operationalised using a video and a click-through guide. To target goals, goal-setting BCTs were selected—in particular, those that focussed on behaviour (e.g. condom use, using lubricant) rather than outcomes (e.g. STI incidence), which may be more distal and less measurable by the user. Users could set time-dependent and event-dependent goals, set reminders, and return to the website to review their goals. Some domains were targeted using a number of written articles (e.g. beliefs about consequences—pleasure), within which multiple BCTs were included. Table 2 details the full range of BCTs selected for each domain.

Once BCTs were selected, they needed to be conceptualised into intervention features. The intervention website was designed to be ‘interactive’, whereby users can input information and get personalised feedback within the website. Resources were focussed on domains which (i) were deemed most important and (ii) afforded themselves to interactive features. For example, an interactive feature which addressed barriers to condom use (‘problem solving’) solicited men’s own problems with condom and then provided tailored information regarding condom types to address those problems. Giving ‘information about health consequences’ was made more engaging by asking users to guess the answers to questions about STI risk, then giving correct responses. A number of videos were also included, which helped to demonstrate the required behaviours and to ensure that the website was engaging.

## DISCUSSION

We have developed a theory- and evidence-based digital intervention to increase condom use in men, using the BCW framework and evidence from the literature, opinions of experts in the field, and data from interviews with the target population. This paper provides an example of how the BCW can be used to create an interactive digital behaviour change intervention, and demonstrates how each step in this process may be carried out.

### The utility of the BCW

The BCW provided a useful and manageable framework for integrating information from multiple sources to design an intervention, especially for organising the information conceptually, for guiding the process of identifying influences on behaviour, and for selecting and delineating standardised methods for targeting those influences.

The advantage of using an integrative theoretical framework, such as the TDF, over a single theory of health behaviour, is that it encompasses multiple explanatory domains and therefore provides a more comprehensive assessment of factors which are important to the target population [20]. This is illustrated in a recent review of theories of behaviour change which found that only three of 83 were integrative and set out to be comprehensive [72, 73]. Models such as the Theory of Planned Behaviour (TPB) [74] present a cognitive model of behaviour (attitudes, perceived norms, and perceived behavioural control predict intentions, which in turn predict behaviour), downplaying impulsive factors in decision-making, which may influence motivation (an important influence on behaviour). Beyond identifying potential influences on behaviour, the BCW also provides structured guidance for determining the content and format of interventions.

While the TDF is helpful in that it offers an extensive list of potential influences on behaviour, some domains were difficult to conceptualise within this context. ‘Goals’ was included as an important domain, but largely as a method of encouraging people to implement the advice given in the website and to target other domains (such as memory, attention, and decision processes). Although the aim of the intervention was to improve intention, this was approached by targeting other domains. The TDF is a framework, rather than a theoretical model, and so does not specify causal pathways between variables. The intervention developer must draw on other evidence and theory to decide and elaborate on potential relationships between variables.

The BCW provides a list of options to select from at each stage of the development process; however, strategic decisions must be made (using personal judgement) regarding the relative importance of different domains and the most appropriate intervention functions and BCTs. Triangulation of evidence from three sources guided decisions; however, where there was conflict, the responsibility for the final decision rested with the research team. The creation of intervention features was not guided by the BCW: imagination and creativity were required to bring intervention functions and BCTs ‘to life’.

### Limitations

The focus of this intervention was to increase condom use in men with inconsistent condom use. As a result, much of the fieldwork work was focussed on barriers to condom use, rather than facilitators. For example, qualitative interviews were conducted with men in sexual health clinics, who had most likely not been using condoms. As a result, the interviews generally focussed on why they were not using condoms rather than what might encourage them to use condoms. This was a limitation, as both barriers and facilitators should be considered when exploring the domains [24]. In addition, some domains were not identified as important during the fieldwork (e.g. optimism). It is difficult to tell whether this is because they are not important in this context or whether there was not sufficient fieldwork conducted to identify them. Including a wider range of data collection measures may have assisted this; interview schedules [75] and questionnaires [27, 76] based on the TDF are available and should be considered in future research.

### Recommendations

The BCW allows intervention developers to define a clear rationale for the specific design and content of interventions. This not only helps the intervention development process but also guides decisions regarding which outcomes to measure when evaluating the intervention through defining the hypothesised ‘active ingredients’ of an intervention.

Specifying the exact nature and content of complex behaviour change interventions is important in order give the possibility to identify which intervention components are the ‘active ingredients’ and to replicate studies [17–19]. This paper explains the rationale for the design and content of the MenSS intervention website and describes the development process. Being explicit about this process contributes to the knowledge about intervention design and development and facilitates the evaluation of intervention effectiveness and the mechanisms of action, helping researchers to avoid reinventing the (potentially ineffective) wheel.

## Footnotes

1 In addition to the steps outlined here, the BCW also includes nine ‘policy’ categories to select from when developing interventions on a wider scale—for example, changing legislation to encourage behaviour change on a population level; however, these were not considered relevant for this (individual level) intervention.
